# Title does matter: a cross-sectional study of 30 journals in the Medical Laboratory Technology category

**DOI:** 10.11613/BM.2020.010708

**Published:** 2020-02-15

**Authors:** Marina Njire Braticevic, Ivana Babic, Irena Abramovic, Anja Jokic, Martina Horvat

**Affiliations:** 1 Department of Laboratory Diagnostics, Dubrovnik General Hospital, Dubrovnik, Croatia; 2 Department for NAT Testing of Blood Donors, Department of Molecular Diagnostics, Croatian Institute of Transfusion Medicine, Zagreb, Croatia; 3 Department of Medical Biology, School of Medicine, University of Zagreb, Zagreb, Croatia; 4 Department of Medical Biochemistry, Haematology and Coagulation with Cytology, University Hospital for Infectious Diseases “Dr. Fran Mihaljević”, Zagreb, Croatia; 5 Department of Medical Laboratory Diagnostics, University Hospital Centre Split, Split, Croatia

**Keywords:** cross-sectional studies, medical laboratory technology, publications, bibliometrics

## Abstract

**Introduction:**

First impression on potential readers is created by the title; therefore, authors should give importance to the title structure. The aim of this study was to establish whether articles created by a smaller number of authors and with shorter, descriptive or declarative titles gain more citations and whether article title length and number of authors correlate to the number of citations.

**Material and methods:**

A cross-sectional study on article citation data for 30 scientific journals published in 2016 in Medical Laboratory Technology field according to Web of Science database was conducted. The type of article, type of title, as well as number of words in the title and number of authors was recorded.

**Results:**

In the group of original articles (N = 2623), articles with declarative titles (N = 336, 13%) showed statistically higher number of citations in multiple comparison analysis when compared to descriptive titles (P < 0.001). No correlation was found between number of citations and title word count (r = 0.07, P < 0.001) nor between number of citations and number of authors in group of original articles (r = 0.09, P < 0.001). Original articles with descriptive titles longer than 15 words or with more than six authors are cited more (P = 0.005 and P < 0.001, respectively).

**Conclusion:**

Based on results of our study, titles do matter. Therefore, authors of original articles might want to consider including their findings in the title and having longer titles.

## Introduction

A part of the professional life of medical experts and scientists includes exchange of information through publishing their own studies and reading other experts’ papers. When searching and reviewing literature, search engines for example Google and online databases such as PubMed/Medline, Web of Science (WoS) and Scopus provide fast and informative response with numerous articles found by title, authors and keywords. More advanced approaches use year span, journal title, type of article, open access, *etc.* (*1*-*3*). With that in mind, keywords and terms in article title are important since they are the same terms indexing services key on (*4*). Working on an article title might not seem crucial, compared to meticulous and often time-consuming manuscript writing. However, given that first impression on potential readers is created by the title, authors should give importance to the title structure because “no abstract can redeem a poorly-crafted title if the reader never clicks on the ‘read abstract’ button” (*5*). Overall, titles should indicate and differentiate a content of the scientific paper from others on the subject (*6*). By recognizing title relevance, some journals in their guidelines advise authors on an informative and concise title, assuming it would help an article to be viewed, downloaded and/or cited (*7*, *8*). Article with higher citation builds author’s reputation and credibility and increases journal’s impact factor (IF) (*9*). Furthermore, a number of scientific papers explored the impact of various article features on article metrics data with title being one of the most studied features (*4*, *9*). Researchers often considered characteristics, such as title’s length and type of title, as relevant for article metrics regarding scientific disciplines and scientific journals. As already mentioned, some journal guidelines for authors, recommend shorter titles and results from some studies are in concordance with this recommendation (*10*, *11*). With quite diversity in study designs regarding selection of data, conflicting results are observed, and studies also reveal quite opposite conclusions that longer titles are getting more citations (*12*, *13*). Studies conducted for longer periods on large amounts of data also show divergence in results of correlation of title length and number of citations regarding fields of science and time period analysed (*14*). Besides length of title, researchers also analysed type of the article title and how they may provoke interest in readers by being interrogative, declarative or descriptive (*14*, *15*). Intriguing article/title feature was also number of authors and how does it correlate to download or citing preferences of readers (*16*, *17*).

Based on previous literature we investigated what findings apply to articles published in journals included in the category Medical Laboratory Technology according to WoS. The aim of this study was to establish whether articles with descriptive or declarative titles gain more citations. In addition, we explored how length of article title and number of authors correlates to the number of citations, respectively, whether article created by a smaller number of authors and with shorter title gain more citations.

## Materials and methods

### Data collection

This cross-sectional study was conducted from August 15^th^ 2018 to February 9^th^ 2019. It included 30 journals in Medical Laboratory Technology category, according to Journal Citation Reports (JCR), which is integrated with the WoS. Web of Science was chosen since all authors had access using institutional sign in *via* AAI@EduHr-the Croatian Research and Education Federation. For each article following data was recorded: journal, issue, title, authors, number of authors, number of citations according to WoS (All Databases), type of article, the number of words in the title and type of title. Year 2016 was chosen for analysis since full citation data were available for that year in the time when the study was conducted.

To collect the data, WoS was searched using the filter: Publication name and Timespan:Custom year range = ‘2016-2016’. Every article was analysed separately. When collecting a number of citations, we recorded results from All Database, which included citations from WoS Core Collection, BIOSIS Citation Index, Chinese Science Citation Database, Data Citation Index, Russian Science Citation Index and SciELO Citation Index.

In the instructions for authors, each journal defines the categories of articles it publishes. According to each journal classification, only original research articles were included in order to minimize confounding by other types of publication. Articles without any journal classification were carefully read and then categorised according to the authors’ assessment.

We also recorded type of title - descriptive, declarative or question, as well as number of words in the title. Descriptive title was considered the one that describes the only subject of the article without revealing conclusion or results. The title was classified as declarative when included not only what the article covered, but also their main conclusion. Finally, question title was considered the one in a form of a question.

### Statistical analysis

To ensure unified word count we used the following formula in Microsoft Excel 2007: number of words in title = LEN(TRIM(cell))-LEN(SUBSTITUTE(cell,” “,””))+1. SUBSTITUTE removes all spaces from the text, and LEN calculates the length of the text without spaces. This number is then subtracted from the length of the text with spaces, and the number one is added to the final result, since the number of words is the number of spaces plus one.

Normality of data was tested using the Kolmogorov-Smirnov test. Data varied from normal distribution is presented as median and interquartile range and as average rank. Kruskal-Wallis test was used to evaluate statistical difference among original articles with different types of titles with *post hoc* analysis in which a subgroup pairwise comparison test was conducted. Mann-Whitney statistical test was used to test the difference between the number of words or number of authors and citations. The correlation was tested with Spearman’s correlation coefficient in order to investigate the relationship between the number of words in the title and number of citations, as well as the number of authors and the number of citations for original articles. Statistical analysis was performed with MedCalc statistical software, version 14.8.1 (MedCalc Software, Ostend, Belgium).

## Results

According to JCR for 2016 there were 30 journals in the category of Medical Laboratory Technology, which published 4323 articles (Table 1).

**Table 1:** Journals and original articles retrieved from Journal Citation Report for 2016 in category of Medical Laboratory Technology

**Journal**	**Original articles, N/total number**
Acta Bioquimica Clinica Latinoamericana	26/90
Advances in Clinical Chemistry	0/40
Annales de Biologie Clinique	20/89
Annals of Clinical and Laboratory Science	75/113
Annals of Clinical Biochemistry	56/107
Annals of Laboratory Medicine	51/117
Applied Immunohistochemistry & Molecular Morphology	93/130
Archives of Pathology & Laboratory Medicine	61/212
Biochemia Medica	23/49
Biomedical Signal Processing and Control	96/113
Biopreservation and Biobanking	58/81
British Journal of Biomedical Science	24/39
Clinica Chimica Acta	293/384
Clinical Biochemistry	172/257
Clinical Chemistry	125/318
Clinical Chemistry And Laboratory Medicine	165/355
Clinical Laboratory	256/330
Clinics in Laboratory Medicine	0/56
Critical Reviews in Clinical Laboratory Sciences	0/30
Cytometry Part B-Clinical Cytometry	45/68
Diagnostic Cytopathology	79/203
Journal of Laboratory Automation (now SLAS Technology)	60/86
Journal of Clinical Laboratory Analysis	192/193
Journal of Cytology	31/63
LaboratoriumsMedizin - Journal of Laboratory Medicine	24/52
Laboratory Medicine	32/65
Pharmaceutical Biology	360/392
Seminars in Diagnostic Pathology	43/43
Therapeutic Drug Monitoring	83/116
Translational Research	80/132

Original articles were analysed according to article type and number of citations (Table 2). A statistical difference in the number of citations among original articles with different types of titles was found (P = 0.029). *Post hoc* analysis showed statistically significant difference between citation number of original articles with descriptive and declarative titles.

**Table 2:** Original article distribution according to type of title and number of citations

**Type of title**	**Number of citations**	**Citation**	**Citation number**
Descriptive (N = 2230)	9864	3 (1 - 6)	1296.0
Declarative (N = 336)	1605	3 (2 - 6)	1411.0
Question (N = 57)	252	3 (1 - 6)	1356.2
Citation is presented as median (interquartile range). The citation number is presented as citation rank.			

Results of title word count and author number comparison with the citation count in the original article group, as well as in the subgroups depending on the type of title are presented in Table 3 and 4. Statistically significant difference was found when comparing citation count for original articles with titles with less than 15 words to titles with more than 15 words (P = 0.007). In addition, statistically significant difference was found when comparing the citation count of original articles with less than six authors to articles with more than six authors (P < 0.001). Similar results were observed in the subgroup of original articles with descriptive titles.

**Table 3:** Comparison of word and author number with citation count in original article titles

	**Original articles (N = 2623)**	**Citation**	**Citation number**	**P**
Word count ≤ 15	1461	3 (1 - 6)	1272.4	0.003
Word count > 15	1162	3 (1 - 6)	1361.9	
Authors ≤ 6	1502	3 (1 - 5)	1259.0	< 0.001
Authors > 6	1121	3 (1 - 6)	1383.1	
Citation is presented as median (interquartile range). The citation number is presented as citation rank. The difference was tested using Mann-Whitney test. P < 0.05 was considered statistically significant.				

**Table 4:** Comparison of word and author number with citation count in original articles with descriptive titles

	**Number of articles**	**Citation**	**Citation number**	**P**
**Original articles with descriptive titles**				
Word count ≤ 15	1267	3 (1 - 5)	1082.0	0.005
Word count > 15	963	3 (1 - 6)	1159.6	
Authors ≤ 6	1290	3 (1 - 5)	1259.0	< 0.001
Authors > 6	940	3 (1 - 6)	1383.1	
**Original articles with declarative titles**				
Word count ≤ 15	155	3 (1 - 6)	166.5	0.721
Word count > 15	181	4 (2 - 6)	170.2	
Authors ≤ 6	177	3 (1 - 5)	159.9	0.086
Authors > 6	159	3 (1 - 6)	178.1	
Original articles with question titles				
Word count ≤ 15	39	3 (1 - 6)	28.5	0.724
Word count > 15	18	3 (2 - 6)	30.1	
Authors ≤ 6	35	3 (2 - 6)	28.2	0.639
Authors > 6	22	3 (1 - 8)	30.3	
Citation is presented as median (interquartile range). The citation number is presented as citation rank. The difference was tested using Mann-Whitney test. P < 0.05 was considered statistically significant.				

No correlation was found between number of citations and title word count (r = 0.09, P < 0.001) nor between the number of citations and number of authors in original articles’ titles (r = 0.10, P < 0.001).

## Discussion

Our study demonstrated a statistically different number of citations between articles with declarative, descriptive and titles in the form of a question, though the same median author number was observed for all three types of titles. When observing rank sum of citation number, we found significant difference between groups of original articles with declarative and descriptive titles with higher number of ranks in the group of articles with declarative title, respectively higher number of citations. This finding could be explained with the fact that articles with declarative title claim their findings, thus making them more interesting to a researcher’s first sight. This is in opposite to findings from a study similar to ours by Jamali *et al.* including 2172 original articles from six journals in the fields of medical and life sciences. They showed that declarative titles are both downloaded and cited less than descriptive titles and commented that might be a little unexpected (*18*). The reason for these different results could be a consequence of varied ways of data collection. This was well explained by Mohebbi *et al.* who examined in their study on 56 journals with 99,838 articles other scientometrics variables, which might have influence on article citations (*19*). They noted that article citation might be influenced by research area, topics, words size, characters, punctuations *etc.*

In this study, no correlation was found between number of citations and the number of words in titles of original articles. This result is in accordance with other authors (*18*, *20*). On the other hand, Habibzadeh *et al.* found positive correlation between the article title’s length and the number of received citations for the group of journals with IF > 10, although the same group of journals was found to have shorter titles than the group with IF < 10 (*13*).

Though we could not observe correlation for a number of original article citations with number of words in the title and number of authors, we compared number of citations for groups of original articles created by the median values for observed features. A significant difference in the number of citations was again found, although the same median was presented in groups of original articles with more or less than 15 words in the title and for articles with more or less than 6 authors. By comparing ranks for groups of original articles subdivided according to type of title, significantly higher rank sum number of citations was found for the group of original articles with descriptive titles with more than fifteen words and a group with more than six authors. This finding agrees with our speculation that declarative titles do catch a researcher’s first sight and more citation regardless of title length and number of authors. On the other hand, in the group of articles with descriptive titles, longer titles and more authors are important for higher number of citations. When comparing our result with literature, heterogeneous results can be found. One of the possible reasons for this discrepancy is that the observed specific relationship between title’s length and number of citations is substantially varying among journals and disciplines. In concordance with results from our study, Hudson elaborates based on his study on 155,500 journal articles in 36 different disciplines that more authors are characteristic of a field of science in comparison to the field of social sciences, arts and humanities and that there is a positive impact of increasing authorship on title length (*21*). Different results are observed if only highly cited journals are analysed, for which positive correlation between citation and shorter titles is present opposed to results for typical journals where positive correlation is lost due to the high heterogeneity of the group (*22*). When observing a number of authors, study by Fox *et al.* explains that positive correlation of citation number and number of authors could be explained by the fact that more authors creating a greater possibility of interaction in researcher’s community, and by that higher number of citations, even higher number of self-citations (*23*). Contrary to this, Ahmed *et al.* in their research on articles from disability related field, present articles with one author as the most cited ones.

This study has limitations that should be considered. Firstly, based on journal categorization, all article types were reduced into six categories for the purpose of analysis. Approximately two thirds of all articles were defined as original and thus included in the analysis. Other types were considered too diverse to be included in further analysis. Using a different grouping could provide different results, as well as different methods of word counting.

Based on the results of our study, titles do matter. Authors who claim their findings in article title or give their article a longer title and with that introduce more Medical Subject Headings (MesH) keywords are getting more reads and therefore more chance to be cited.
